# Toe Separators as a Therapeutic Tool in Physiotherapy—A Systematic Review

**DOI:** 10.3390/jcm13247771

**Published:** 2024-12-19

**Authors:** Hanna Krześniak, Aleksandra Truszczyńska-Baszak

**Affiliations:** 1 Doctoral School, Jozef Pilsudski University of Physical Education, 00-968 Warszawa, Poland; 2 Faculty of Rehabilitation, Jozef Pilsudski University of Physical Education, 00-968 Warszawa, Poland; aleksandra.truszczynska@awf.edu.pl

**Keywords:** foot deformities, rehabilitation, toe separators, toe spacers, hallux valgus

## Abstract

**Background:** Deformities of the foot represent a significant clinical problem. Toe separators constitute an available tool used in various forms of conservative treatment, primarily used for the correction of hallux valgus, but also for improvement in the condition of neurological patients, e.g., after a stroke, or to treat dermatological problems. The goal of this systematic review is to critically assess the current scientific literature on the application of toe separators as a therapeutic intervention in physiotherapy. **Methods:** A systematic search was conducted across several electronic databases, such as PubMed, Science Direct, and Web of Science. The review included randomized controlled trials, quasi-experimental studies, and observational studies that explored the use of toe separators in physiotherapeutic interventions. Two independent reviewers evaluated all search results to determine eligible studies and assess their methodological quality. **Results:** A total of 1020 studies were found through the database search. Out of these, 10 studies met the inclusion criteria and were incorporated into the review. The sample sizes of the selected studies varied from 9 to 90 participants. In the majority of the studies, the methodological quality was not mentioned, and a frequent lack of information was noted. Based on a literature analysis, separators were primarily used as a tool for the correction of hallux valgus, but there are also promising results for use in neurology and dermatology as well as affecting the lower leg muscles during gait. **Conclusions:** The use of toe separators can be a valuable tool for the conservative treatment of hallux valgus and the associated deformities. The research so far varies in describing the type, material, and method of the application of the separators. Studies showed a variety of applications as well as variation in the use of materials. Further research is needed to establish the effectiveness of toe separators in foot disorders more precisely.

## 1. Introduction

Toe separators, a simple yet effective orthotic, have gained attention as a potential intervention for various foot and lower limb conditions. They are typically made of soft silicone or foam, and they are designed to create space between the toes thereby addressing alignment issues, improving muscle function, and alleviating pain. Toe separators are primarily recommended for toe deformities, especially hallux valgus. The hypothesis of this study is that separators can be effective in treating hallux valgus toe deformity but can also be helpful in other, co-occurring deformities.

During gait, three parts of our foot are involved in walking: the rearfoot, midfoot, and forefoot. Each of these plantar regions plays a specific biomechanical role in shock absorbing and helping the forward propulsion of the body. Many studies have investigated the shock absorbing capabilities of these areas, examining various factors that influence plantar compression and pressure, such as the soft tissues of the foot, the medial longitudinal arch, and the posture of the foot [1]. However, the forefoot is the area we have the most influence on, which also makes it most prone to deformation caused by improper footwear.

Forefoot disorders are common in the general population and can have a substantial effect on people’s daily activities and their physical function [2,3]. These conditions are often linked to factors such as wearing poorly fitted or high-heeled shoes, abnormal foot alignment, and foot joint disorders [4]. Typical forefoot disorders include metatarsalgia, hallux valgus, hallux limitus/rigidus, deformities of the lesser toes (such as hammer, claw, and mallet toes), and Morton’s neuroma (interdigital neuroma) [5].

Hallux valgus (HV) is a common foot deformity with its prevalence varying across different studies. It is estimated to affect 2–4% of the general population [6], with rates rising up to 23% in adults and 35.7% in the elderly [7]. Cai et al. estimated the globally pooled prevalence and incidence of hallux valgus to be 19% [8].

The development of HV is multifactorial. It is often linked to genetic predisposition and may be influenced by factors such as hypermobility, wearing tight or high-heeled shoes, and obesity. Hecht and Lin reported that women are diagnosed with this condition up to 15 times more often than men [6].

Significant factors influencing hallux valgus included a larger first intermetatarsal angle, a longer and rounded first metatarsal head, and lateral sesamoid displacement. Clinical factors, such as first ray mobility, pes planus, and footwear, showed less definitive links to HV in a systematic review conducted by Nix et al. [9].

There is a notable discrepancy between self-reported and radiographic evaluations of HV, which diminishes as the condition worsens. Relying on self-assessment may underestimate the prevalence of HV [10].

Managing such disorders commonly involves exploring conservative treatment methods before considering surgical intervention. Such conservative treatment methods include using corrective footwear, insoles or orthotics (like pads or supports), oral medications, and corticosteroid injections [7]. However, despite the common occurrence of forefoot disorders, there is no research exploring the effectiveness of conservative treatments.

Although, as literature shows, toe separators are primarily intended for the conservative treatment of hallux valgus, this systematic review aims at verifying the possibility of using toe separators in relation to other disorders [11].

The goal of this systematic review is to critically assess the current scientific literature on the application of toe separators as a therapeutic intervention in physiotherapy.

## 2. Materials and Methods

### 2.1. Search Strategy

A comprehensive search was carried out across several electronic databases, such as PubMed, ScienceDirect, and Web of Science, to conduct this review. The following are the keywords used in the search: toe separators, toe spreaders, toe spacers, toe separators hallux valgus. Research databases were searched one by one with the use of all keywords and thus articles related to the topic of toe separators were selected. Words were typed without additional operators and searched by topic. Additionally, the reference lists of the identified articles were screened to ensure a comprehensive inclusion of relevant studies. The search was restricted to articles published in English.

### 2.2. Study Selection and Data Extraction

**Inclusion criteria:** This review encompassed randomized controlled trials, quasi-experimental studies, and observational studies that investigated the use of toe separators in interventions. Studies focusing on various foot and lower limb conditions, such as hallux valgus, plantar fasciitis, and metatarsalgia, were also considered.

**Exclusion criteria:** Non-English articles, lack of accessible article content or even abstracts alone, topics not related to toe separators, use of other types of orthoses (e.g., orthoses to abduct the hallux valgus, not acting as toe separators), descriptions of surgical procedures, and articles describing other topics not related to toe separators.

This systematic review was conducted following the guidelines outlined in the Preferred Reporting Items for Systematic Reviews and Meta-Analyses Protocols (PRISMA-P). These guidelines provide a structured framework for the transparent and comprehensive reporting of systematic review protocols, ensuring the reproducibility and quality of the review process [12]. Data extraction was carried out using a standardized template. The following information was gathered from each included study: study design, participant details (such as sample size, age, and gender), intervention specifics (including the type of toe separators used, duration, and frequency), outcome measures, and reported results.

### 2.3. Quality Assessment

The included studies underwent a quality assessment using established evaluation tools, such as the PEDRO scale. Relevant information was extracted, including study design, participant demographics, intervention specifics, outcome measures, and reported findings [13,14].

Additionally, we utilized the PICO (Population, Intervention, Comparison, Outcome) framework to guide the selection of the studies. This framework helps to define key elements of our review, including the target population (patients with foot and lower limb conditions), the intervention of interest (toe separators), the comparison groups (if applicable), and the desired outcomes (e.g., pain reduction, functional improvement) [15] (Table 1).

**Table 1:** PICO framework.

P	I	C	O
Population/Problem	Intervention	Comparison	Outcome
patients with foot and lower limb conditions	toe separators	other treatment	pain reduction, functional improvement, improvement in hallux valgus angle

Through this systematic review, we aimed to provide physiotherapists, researchers, and healthcare professionals with an evidence-based understanding of the potential benefits and limitations of toe separators as a therapeutic tool.

Two independent reviewers examined the titles and abstracts of the identified articles to determine their eligibility for inclusion. Full-text versions of potentially relevant studies were subsequently retrieved and evaluated for final inclusion. Any discrepancies between the reviewers were resolved through discussion and consensus.

### 2.4. Data Synthesis and Analysis

Given the expected variability among the studies included, a meta-analysis was not possible. Instead, a narrative synthesis of the results was conducted. The findings were organized and presented based on the specific foot and lower limb conditions studied, with a focus on the outcomes and conclusions reported in each study.

### 2.5. Protocol

In addition to conducting a systematic review, this study also followed the best practices of transparency and reproducibility by registering the protocol on protocols.io (Protocol Integer ID: 92095). It is an online platform that allows researchers to openly share and document their research protocols, ensuring transparency and facilitating collaboration within the scientific community [16]. Registering the protocol aimed at increasing the study’s credibility by outlining the research methodology, including the search strategy, inclusion criteria, data extraction, and quality assessment tools.

This registry serves as a comprehensive record of our study design, enabling other researchers to replicate or build upon our work. By adhering to the principles of open science and registering our systematic review protocol, we aimed at contributing to the growing body of transparent and reproducible research in the field of physiotherapy [17].

## 3. Results

### 3.1. Systematic Review

**Figure 1:**
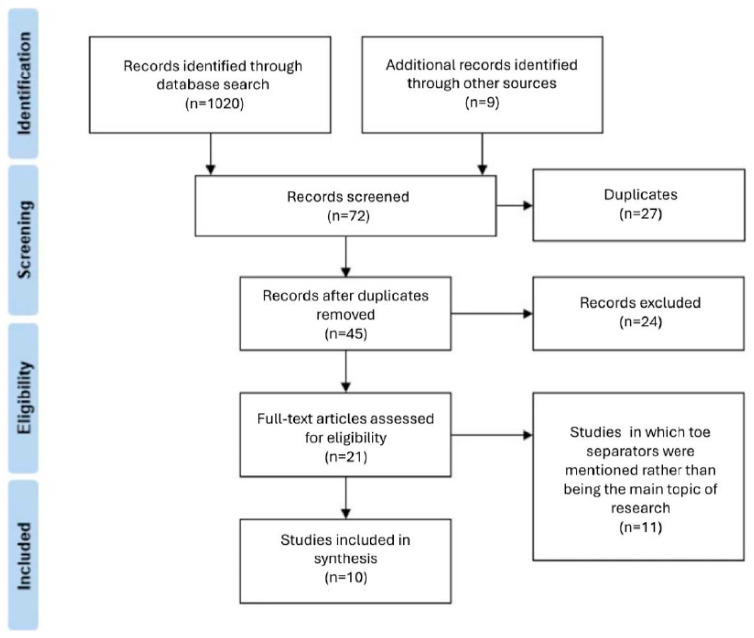
Summary of research search and selection process in PRISMA-P framework.

A total of 1020 articles were identified through systematic search, and after applying the inclusion and exclusion criteria, 72 were deemed eligible for this review. After removing duplicates, 45 items remained related to the topic of toe separators. There were 21 items that did not meet the inclusion criteria, of which 11 mentioned the use of separators as a possible application (e.g., for the treatment of hallux valgus deformity) while 10 investigated the impact of toe separator use [shown in Figure 1].

He selected articles encompassed a diverse range of foot and lower limb conditions, including hallux valgus, plantar fasciitis, and metatarsalgia, as well as other publications that mentioned or analyzed the use of toe separators.

The studies included in this review used a range of study designs, such as randomized controlled trials (RCTs), quasi-experimental studies, and observational studies. Sample sizes varied, with a minimum of 9 participants and a maximum of 90. The interventions involving toe separators varied in terms of duration, frequency, and specific application techniques. Some studies utilized toe separators as a standalone intervention, while others incorporated them as part of a comprehensive physiotherapy treatment plan. The duration of intervention ranged from immediate to long-term effect in order to assess the sustained effects of toe separators. Importantly, the articles did not always specify whether the study involved separators for five toes or only for one toe: between the big toe and the second toe.

Outcome measures used in the included studies were diverse, reflecting the multifaceted nature of foot and lower limb conditions. Commonly assessed outcomes included pain intensity, foot alignment, muscle strength, functional mobility, and patient-reported outcomes. Objective measurements, such as gait analysis and imaging techniques, were also employed in some studies to provide a more comprehensive evaluation of the effects of toe separators.

### 3.2. Non-Operative Management

Some of the articles from the study did not directly examine the effect of toe separators, but they were mentioned as an element of conservative treatment for various forefoot disorders. Most of the articles where the use of toe separators was mentioned involved the correction of hallux valgus.

Many authors advised performing a non-operative treatment before surgery [18,19,20,21]. Leucht et al. stated that non-operative treatments, such as custom orthotics and shoes with a wide toe box, could be effective. For lesser toe deformities, they recommended using silicone sleeves and toe separators to prevent painful calluses [18]. Similar findings were made by Varacallo et al., who also recommended the use of shoe modifications, metatarsal pads, toe separators, and protective sleeves for managing lesser toe deformities. Additionally, the authors emphasized the importance of physiotherapy support, which facilitates gait re-education and reduces the risk of metatarsalgia-related symptoms [19]. Aebischer et al. stated that the initial treatment for symptomatic bunions should focus on non-surgical approaches, with accommodative footwear being a key component. They found some evidence supporting the use of orthotics, toe separators, splints, and braces, which may offer symptomatic relief and reduce pain in certain patients [20]. Ledoux et al. noted that a variety of non-surgical treatments, including foot orthoses, night splints, toe separators, manual therapy, and foot exercises, could be explored before opting for surgical correction of hallux valgus (HV), though evidence for their effectiveness remains limited. They also highlighted the need for long-term prospective studies to examine the risk factors for HV and the efficacy of non-surgical treatments [21].

### 3.3. Postoperative Management

Toe separators are also applicable in the postoperative treatment of hallux valgus. Arbab et al. recommended postoperative management based on bone correction, advising a corrective bandage for 3 weeks following joint-preserving procedures, followed by a hallux valgus orthosis at night and toe separators for 3 months [22]. A similar approach was suggested by Waizy et al., who recommended 4 weeks of postoperative dressing, control after 6 weeks, and the use of a night orthosis and toe separators for additional 6 weeks [23]. In contrast, Ponzio et al. compared post-surgery use of taping and toe separators, finding no radiographic benefit from taping after hallux valgus correction [24].

### 3.4. Skin Problems

Günal et al. reported on ten cases of stage I and four cases of stage II onychocryptosis, all treated by placing toe separators between the first and second toes. The condition healed in approximately three weeks [25].

Toe separators can also be used for DFUs (diabetic foot ulcers) and should be considered depending on the ulcer location [26].

Gupta et al. proposed that separators could be made individually by patients or clinicians by using hot glue. This helps to reduce the cost and difficulty of fitting the separators. The authors looked at ways of treating interdigital infections particularly in patients with obesity, arthritis, diabetes, and other orthopedic problems. The primary approach to treating interdigital infections is maintaining dryness in the web spaces, which can be achieved by keeping the toes apart [27].

### 3.5. Toe Separators–Narrative Synthesis

Articles focused mainly on toe separators can be divided into three categories: the treatment of hallux valgus, intervention in patients with neurological symptoms such as after a stroke, and pure muscle tension analysis with the use of toe separators [Table 2 summarizes the articles taken for analysis, with the presented: author, material, methods, results].

### 3.6. The Correction of a Hallux Valgus–Meta-Analysis

The most common usage of toe separators described in the literature was the correction of hallux valgus. The network meta-analysis by Ying et al. found that a combination of exercise, toe separators, night splints, and dry needling was likely the most effective option for reducing the hallux valgus angle (HVA) and intermetatarsal angle. Additionally, toe separators improved patients’ subjective well-being.

Authors concluded that it was crucial to implement a multidisciplinary approach towards a patient with hallux valgus, considering their individual clinical condition, needs, and preferences [7].

Kwan et al.’s meta-analysis found that orthoses with a toe separator were most effective in correcting the hallux valgus (HV) angle (standardized mean difference: 0.50, 95% CI: 0.189 to 0.803). HV is often caused by prolonged training by athletes. They concluded that it can be managed conservatively with foot orthoses being a popular non-surgical option. Dynamic orthoses tend to be preferred over static ones due to the better fit, comfort, and appearance. Both types, especially static ones with toe separators, were shown to reduce the HV angle. The use of orthoses with toe separators can reduce the HV angle by 2.1° to 5.79° and alleviate pain by improving big toe alignment and relieving stress on ligaments and bones [28].

### 3.7. Insole and Single Toe Separator in Patients with Hallux Valgus

A factor indicated in many publications on the improvement of the condition of patients with hallux valgus was the use of orthotics. Dissaneewate et al. conducted a study in which 23 patients with hallux valgus were randomly assigned to use either a prefabricated toe separator or a custom insole. The plantar pressure distribution was measured while walking with the devices after one month of use. The results showed significant reductions in peak pressures and the pressure–time integral in the middle and lateral forefoot areas for those using the custom insole (64.28 kPa and 28.97 kPa s, respectively, in the middle forefoot; 54.03 kPa and 22.30 kPa s, respectively, in the lateral forefoot) compared to the ones using a toe separator. One month the custom insole proved more effective in reducing plantar pressure than the toe separator in individuals with hallux valgus [29].

Similar results were obtained previously by Tang et al. while analyzing the effect of an orthotic with a built-in separator on one toe. In an uncontrolled intervention study involving 17 patients with painful hallux valgus, a new total contact insole with a fixed toe separator was applied. The average reduction in the hallux valgus angle was 6.5° ± 3.8° following the use of the insole (*p* < 0.001). Pain, as measured on the NRS-11 scale, decreased from 4.06 ± 2.8 to 0.88 ± 1.17 immediately after insole application (*p* < 0.001), with a further reduction to 0.42 ± 0.67 (*p* = 0.002) three months later for the 12 patients who completed the study. Walking ability also improved by at least one grade after three months of using the insole (*p* = 0.002). All patients tolerated the insole well and the total contact insole with the fixed toe separator effectively reduced pain, improved walking ability, and decreased the hallux valgus angle [30].

Tehraninasr et al. studied the effect of an insole with a toe separator on 30 female patients (ages 19–44) with painful bunions, a hallux valgus angle of 35° or less, and an intermetatarsal angle of 15° or less. The hallux valgus and intermetatarsal angles were measured radiographically and foot pain intensity was assessed using the VAS before the intervention and after a 3-month follow-up. The study found that the insole with the toe separator was an effective treatment for reducing pain in patients with hallux valgus deformity, while the night splint showed no significant effect on pain relief. Although neither device corrected the underlying deformity, both prevented further progression of the condition in the two groups [31]. However, in the aforementioned study, only a single separator between the big toe and the second toe was analyzed with no mention of separators for all five toes.

### 3.8. Effect of Toe Separators on Hallux Valgus

Toe separators were recommended as a treatment for hallux valgus [7,32,33]. However, they do not fit every type of deformity. Chadchavalpanichaya et al. conducted a study to evaluate the effectiveness of custom molded room temperature vulcanized silicone toe separators. The study involved 90 patients with moderate hallux valgus, who were randomly assigned to two groups. The study group used a toe separator for 6 h per night for 12 months, while both groups received proper foot care and shoes and were allowed to continue any drug treatments. After 12 months, the study group showed a significant reduction in the hallux valgus angle (3.3° ± 2.4°), while the control group experienced an increase (1.9° ± 1.9°), with statistically significant differences between the two groups (*p* < 0.05). Additionally, the study group reported decreased hallux valgus pain. The custom molded silicone toe separators effectively reduced the hallux valgus angle and pain with no serious complications [32].

### 3.9. Foot Mobilization and Exercise Program Combined with Toe Separators

In a randomized clinical trial by Abdalbary et al., similar improvements in pain reduction and function were observed. The study assessed the effectiveness of a 3-month intervention combining foot mobilization and exercise with a toe separator in patients with moderate hallux valgus. A total of 56 women were randomly assigned to either the intervention group (36 sessions over 3 months) or a waiting list control group. Those who received the treatment showed significant improvements in pain reduction, AOFAS scores, ankle range of motion, hallux plantar flexion and abduction strength, toe grip strength, and radiographic angular measurements in comparison to the control group. Both groups were respectively evaluated at 3 months and 1 year post-intervention (*p* < 0.001 for all comparisons). Foot mobilization and exercise combined with a toe separator proved to be an effective treatment for moderate hallux valgus [33].

### 3.10. Effect of Toe Separators on Shin Muscle Activation

Disorders of the lower leg are a common problem faced by clinicians, and complaints related to this area may have a root cause in the foot system [34,35]. The shin muscles, including the anterior tibialis and posterior tibialis muscles that are attached to the foot, play an important role in preventing the collapse of the medial longitudinal arch of the foot [36]. Anterior tibialis plays an important role as the flexor of the ankles and the knees and is highly activated during the stance period [37]. Peroneus longus has a complex anatomical structure, and its extended pathway can lead to symptoms affecting the lower leg, ankle, hindfoot, and the plantar region. The impact of calf muscles is significant, as they can affect the entire lower limb [38]. The metatarsophalangeal (MTP) joint has an important function during walking so the correct alignment of the big toe is essential as its incorrect position can negatively affect gait biomechanics [39].

Lee et al. explored how different materials used for toe separators can affect muscle activity during walking. EMG activity of the tibialis anterior (TA) and peroneus longus (PL) muscles (%RVC) was measured on 20 healthy participants with and without toe separators made from two different materials (soft and hard). When walking with soft toe separators, TA activity was significantly increased, whereas it was reduced with hard toe separators. PL activity was notably decreased with soft toe separators. These changes in TA and PL muscle activity may be due to the medio–lateral foot support provided by the toe separators during walking [40].

### 3.11. Use of Toe Separators as Tool in Neurology

Claw toe is a structural foot deformity that may occur after a stroke and cause functional changes that affect both the lower limbs and balance.

For this reason, researchers studied the use of toe separators as a tool that can potentially correct the alignment of toes and thus the whole foot. The first studies investigating the effect of separators on gait in people with neurological problems were initiated by de Saca et al. as early as 1994, when they carried out a randomized study on 18 adults with hemiparesis secondary to supraspinal lesion. They assessed whether the use of toe separators immediately inhibits the tonic toe flexion reflex (TTFR) and if it affects temporal-distance gait characteristics, plantar surface contact, or muscle activity in the limb displaying the TTFR. The study included inter-conditional comparisons during both the standing and gait phases including four conditions for each phase (shoe off, toe separators off/on, shoe on, toe separators off/on). The measures taken included ink footprint gait analysis and integrated electromyography from the limb exhibiting the TTFR. The results showed a significant reduction in the presence of the TTFR with the use of the toe separators. Additionally, velocity and cadence were notably increased with the toe separators. The findings suggested that the toe separators may be a beneficial treatment for improving gait [41].

Several years later, Chiong et al. revisited the use of toe separators in neurological patients, conducting a single-blind, randomized controlled pilot trial with nine ambulatory participants, who had tonic toe flexion reflexes for more than six months after a stroke. They assessed the long-term effects of toe separators on gait characteristics, pain, and activity levels. The measures included gait speed, plantar surface contact area, pain assessed via the Visual Analog Scale (VAS), the Berg Balance Scale, the Modified Ashworth Scale, activity levels measured with a pedometer, and compliance tracked through a logbook. The intervention group wore customized toe spreaders combined with sport sandals for walking during a six-month-period, whereas the control group received standard physiotherapy. There were no significant differences between the groups. However, all participants in the intervention group used the toe separators less than 50% of the day, indicating suboptimal compliance. The use of toe separators did not result in significant improvements in any of the outcomes of the intervention. Authors suggested that the studies should be conducted in groups of at least 56 participants and that there should be strategies in place that would increase compliance with the instruction regarding the systematic use of toe separators [42].

The most recent publication related to this topic was conducted by Lee et al., who examined the effects of a toe separators on foot pressure and gait in 25 chronic stroke patients. Using the Gaitview AFA-50 and GAITRite systems, they measured plantar pressure distribution and gait with and without toe separators. Although, the average and rear foot pressures increased with the use of the toe separators, these changes were not statistically significant in post hoc tests. However, all gait variables showed significant improvement when toe spreaders were used. The study suggested that toe separators may enhance overall gait in chronic stroke patients [43].

**Table 2:** Articles analyzed, hallux valgus (HV), hallux valgus angle (HVA), the intermetatarsal angle (IMA), Visual Analog Scale (VAS).

Authors	Aim	Material	Methods	Results
Ying et al. [7]	Comparisons of conservative treatments for hallux valgus: a systematic review and network meta-analysis	participants with HV, conservative treatments, HVA, IMA, VAS, Foot Function Index	searching PubMed, EMBASE, MEDLINE, OVID, and CINAHL	combination of exercise and toe separator, night splints, and dry needling helps with HV
Kwan et al. [28]	Hallux valgus orthosis characteristics and effectiveness: a systematic review with meta-analysis	HV orthosis design	PubMed, Scopus, Cinahl, and Medline searching PubMed, Scopus, Cinahl, and Medline	dynamic orthoses, and static orthoses with a toe separator help to reduce the HVA ~ 2.1° to 5.79° among
Dissaneewate et al. [29]	Comparison between the plantar pressure effects of toe separators and insoles in patients with hallux valgus at a one-month follow-up	23 HV patients	prefabricated toe separator vs. customized insole	customized insole was more effective in plantar pressure reduction than the toe separator for a hallux deformity
Tang et al. [30]	Effect of an orthotic with a built-in separator on one toe	17 painful HV	application of insole with fixed toe separator	reduced pain, improved walking ability and HVA
Tehraninasr et al. [31]	Effects of insole with toe-separator and night splint on patients with painful hallux valgus	30 females with HV	the insole and toe separator vs. night splint	insole with toe separator reduces pain, not effective improvement of HVA
Abdalbary et al. [33]	Foot mobilization and exercise program combined with toe separator	56 females with moderate HV	36 sessions over 3 months vs. no intervention	improvement in pain, AOFAS scores, ankle range of motion, hallux plantarflexion and abduction strength, toe grip strength, and radiographic angular measurements
Lee et al. [40]	Immediate effect of the toe spreader on tibialis anterior and peroneus longus muscle activities	20 healthy participants	soft vs. hard toe spreaders EMG activities (%RVC) tibialis anterior (TA) and peroneus longus (PL)	TA activity was increased by the soft toe spreader and was decreased by the hard toe spreaders PL was decreased by the soft toe spreader
de Saca et al. [41]	Immediate effects of the toe spreader on the tonic toe flexion reflex	18 adults with hemiparesis secondary to supraspinal lesion	ink footprint gait analysis and EMG	velocity and cadence were increased significantly by use of the toe spreader
Chiong et al. [42]	The effects of toe spreader in people with overactive toe flexors post stroke	9 ambulatory participants with tonic toe flexion reflex more than 6 months post stroke	customized toe spreader during rehabilitation vs. standard rehabilitation gait, VAS, Berg Balance Scale, Modified Ashworth Scale, pedometer, and compliance via logbook	no significant differences between the groups, but intervention group used the toe spreader less than 50% of the days
Lee et al. [43]	Effects of toe spreader on plantar pressure and gait in chronic stroke patients	25 chronic stroke patients	with vs. without a toe spreader plantar pressure distribution and gait	gait significantly improved when the toe spreader was used the average and rear foot pressures increased when a toe spreader was used (not significant in post hoc)

## 4. Discussion

Overall, the findings from the included studies stated that toe separators have the potential to be an effective therapeutic tool in physiotherapy interventions for various foot and lower limb conditions. Several studies reported significant improvements in pain reduction, foot alignment, muscle strength, and functional outcomes following the use of toe separators. However, it is important to note that the level of evidence varied across studies as some demonstrated stronger methodological rigor than others.

There were some difficulties in determining the exact target group for whom toe separators would be useful. This may be partly due to the fact that deformities in the foot are often co-occurring problems, for example, a flexible flat foot along with hallux valgus [44,45,46,47].

However, according to Suh et al., in adult patients, pes planus does not show a significant correlation between severity or recurrence of hallux valgus, nor radiographic findings or clinical assessments [48].

Moreover, the shape of the foot, including its arches, is influenced by more factors, such as the work of gluteal muscles and not just the muscles of the foot itself. Goo et al. suggested that strengthening the gluteus maximus alongside exercises correcting foot pronation can effectively restore normal gait [49].

Furthermore, strengthening of the toe abductor muscle is facilitated by the use of toe separators.

In contrast, another study showed a positive effect of strengthening exercises on the posterior tibialis muscle combined with stretching the iliopsoas muscle [50].

Despite the promising results, it is worth mentioning that a few studies reported limited or inconclusive findings regarding the effectiveness of toe separators. Factors such as participant characteristics, condition severity, and adherence to the intervention may have influenced the outcomes observed in these studies.

Studies on the treatment of hallux valgus often do not specify additional deformities in the foot that can come along with this condition, such as Metatarsus Adductus [51].

Toe separators have drawn the attention of clinicians and researchers not only because of their physical benefits in increasing foot health and comfort but also because of their potential impact on overall well-being. In the context of adults with disabilities, where the incidence of poor mental health is prevalent, implementing moderate-to-vigorous intensity physical activity into daily routines could serve as a catalyst for improvement [52]. Additionally, incorporating interventions like toe separators may serve as a simple yet effective means of fostering engagement in physical activity, potentially leading to healthier and happier lives.

### 4.1. Limitation

One of the limitations of this systematic review is the heterogeneity of the included studies, which varied in terms of study design, sample size, and outcome measures. Many of the selected studies lacked detailed information on the methodological quality and the specific materials used for the toe separators, making it difficult to draw definitive conclusions regarding the most effective type and application method. An important problem is the confusion of terminology: toe separator is the term used in the literature for both a single separator between the big toe and the second toe, and separators for five toes. In our work, we have consistently used the terms “toe separators” to refer to five-toe separators, and “single toe separator” to emphasize a single separator. In addition, the wording separators/spreaders/spacers is used interchangeably in the scientific literature. Finally, the review was limited to articles published in English, potentially excluding relevant studies in other languages. Further high-quality, well-designed studies are necessary to better understand the efficacy and optimal use of toe separators for various foot conditions.

### 4.2. Perspectives

The findings of this systematic review highlight the potential value of toe separators as a non-invasive treatment option for various foot deformities, particularly hallux valgus. However, the variability in study designs, sample sizes, and the materials used in the interventions emphasize the need for further standardization in both research and clinical practice. Future studies should focus on establishing clearer protocols regarding the types of toe separators (single or for five toes) and their materials and application methods in order to better assess their efficacy.

Long-term, high-quality randomized controlled trials with larger and more diverse participant groups (for example, people with flexible flat feet) are necessary in order to provide stronger evidence for the effectiveness of toe separators across different conditions. Additionally, exploring the biomechanical mechanisms of toe separators and their impact on gait and foot function will be crucial in understanding their broader therapeutic potential. Ultimately, a more solid evidence base will help clinicians make decisions on incorporating toe separators into their treatment protocols.

## 5. Conclusions

In conclusion, the available evidence indicated that toe separators are promising as a therapeutic tool in physiotherapy interventions in foot and lower limb conditions. Most reports consider toe separators for treating hallux valgus, both conservatively and as maintenance treatment after surgery. In addition, there are also studies examining the effect of separators on neurological and dermatological patients, where separators may also have an adjunctive function in the therapy process, which is in line with the original hypothesis. However, further high-quality research is warranted to establish the optimal application techniques, duration, and long-term effects of toe separators. Future studies should also consider standardized outcome measures and larger sample sizes in order to enhance the generalizability of the findings.
